# Deciphering the Link: Correlating REM Sleep Patterns with Depressive Symptoms via Consumer Wearable Technology

**DOI:** 10.3390/jpm14050519

**Published:** 2024-05-14

**Authors:** Cătălina Angela Crișan, Roland Stretea, Maria Bonea, Vadim Fîntînari, Ioan Marian Țața, Alexandru Stan, Ioana Valentina Micluția, Răzvan Mircea Cherecheș, Zaki Milhem

**Affiliations:** 1 Department of Neurosciences, Psychiatry and Pediatric Psychiatry, Faculty of Medicine, Iuliu Hațieganu University of Medicine and Pharmacy, 400012 Cluj-Napoca, Romania; ccrisan@umfcluj.ro (C.A.C.); bonea.maria@umfcluj.ro (M.B.); imiclutia@umfcluj.ro (I.V.M.);; 2 Clinical Hospital of Infectious Diseases, 400348 Cluj-Napoca, Romania; 3 SC STEEPSOFT AI SRL, 505600 Sacele, Romania; 4 Automatics and Computers Doctoral School, Politehnica University of Bucharest, 060042 Bucharest, Romania; 5 Clinical Emergency Hospital for Children, 400370 Cluj-Napoca, Romania; 6 Department of Public Health, College of Political, Administrative and Communication Sciences, Babeș-Bolyai University, 400294 Cluj-Napoca, Romania; razvan.chereches@ubbcluj.ro

**Keywords:** REM sleep, depression, Apple Watch, personalized management

## Abstract

This study investigates the correlation between REM sleep patterns, as measured by the Apple Watch, and depressive symptoms in an undiagnosed population. Employing the Apple Watch for data collection, REM sleep duration and frequency were monitored over a specified period. Concurrently, participants’ depressive symptoms were evaluated using standardized questionnaires. The analysis, primarily using Spearman’s correlation, revealed noteworthy findings. A significant correlation was observed between an increased REM sleep proportion and higher depressive symptom scores, with a correlation coefficient of 0.702, suggesting a robust relationship. These results highlight the potential of using wearable technology, such as the Apple Watch, in early detection and intervention for depressive symptoms, suggesting that alterations in REM sleep could serve as preliminary indicators of depressive tendencies. This approach offers a non-invasive and accessible means to monitor and potentially preempt the progression of depressive disorders. This study’s implications extend to the broader context of mental health, emphasizing the importance of sleep assessment in routine health evaluations, particularly for individuals exhibiting early signs of depressive symptoms.

## 1. Introduction

Sleep, a reversible and complex physiological state, is characterized by distinct cerebral electrical activities, marking a stark contrast from wakefulness. Once the body transitions from wakefulness, it enters a sleep cycle that alternates between non-rapid eye movement (NREM) and REM sleep. Each phase has unique characteristics and implications for brain function and overall health. REM sleep, in particular, is synonymous with the dream state and is marked by rapid eye movements, a mixed-frequency electroencephalographic pattern, and muscle atonia. This muscle atonia during REM sleep is not merely a physiological curiosity but serves a vital protective role. It prevents the physical enactment of dreams, safeguarding the body from potential harm due to involuntary movements that may occur during vivid dreams [1,2,3,4]. Sleep is an essential human requirement, critical for maintaining overall health and well-being. Adequate nocturnal sleep positively influences physical and mental health, thereby enhancing life quality. During sleep, physiological processes engage in maintaining and restoring physical and mental states, playing a key role in disease prevention and supporting growth and development, particularly in children. Consequently, well-being and wakeful performance are heavily influenced by nocturnal activities. Ensuring sufficient sleep, aligned with circadian rhythms, is vital for numerous biological processes and systems [5,6,7].

Several studies have highlighted a significant interaction between cholinergic and monoaminergic neurons located in the brainstem. This interaction forms an intricate intercellular network crucial for regulating the initiation of REM sleep [8,9]. Within this complex, cholinergic neurons, known for their role in arousal and alertness, dynamically interact with monoaminergic neurons, which are integral in mood regulation and sleep–wake transitions. Central to this process are biogenic amines, particularly norepinephrine and serotonin, which play pivotal roles in the generation and maintenance of sleep. These neurotransmitters are actively involved in initiating various sleep phases, yet their levels are notably reduced during REM sleep. This reduction in neurotransmitter activity during REM sleep is a subject of keen interest in sleep research, as it presents a unique neurochemical state within the sleep cycle [10,11]. Moreover, the balance and functioning of norepinephrine and serotonin systems are critical for normal sleep patterns. Imbalances or disturbances in these systems can lead to abnormalities in REM sleep. Such dysregulation is often observed in various psychiatric conditions, notably endogenous depression and anxiety disorders [12,13,14].

These findings suggest a potential link between the neurochemistry of sleep and mental health disorders, indicating that the dysregulation of neurotransmitters like norepinephrine and serotonin during REM sleep could contribute to or exacerbate these conditions. Understanding this complex relationship offers valuable insights into the neurobiological mechanisms underlying both sleep and psychiatric disorders, paving the way for more targeted and effective treatments.

Depression is a common and serious illness that profoundly impacts individuals. This condition is well-defined in the Diagnostic and Statistical Manual of Mental Disorders (DSM). The DSM categorizes an episode of depression as a period of at least two weeks where a person experiences at least five of the outlined symptoms, with at least one being either a depressed mood or a loss of interest or pleasure in previously enjoyed activities. Other symptoms include significant weight loss or gain, insomnia or hypersomnia, psychomotor agitation or retardation, fatigue or loss of energy, feelings of worthlessness or excessive guilt, and cognitive impairments [15].

Depression represents a significant public health issue due to its prevalence and the severity of its symptoms. Globally, it is estimated that about 3.8% of the population suffers from depression. This includes 5% of adults, with a slightly higher prevalence in women (6%) compared to men (4%). Among older adults, those over 60 years, the prevalence is about 5.7%. It is estimated that approximately 280 million people worldwide are affected by depression [16]. The impact of depression extends beyond the individual, affecting families, workplaces, and communities. It can lead to decreased productivity, increased absenteeism, and can strain relationships. Depression also has a significant economic impact, with substantial costs associated with healthcare and a loss of work productivity.

Treatment for depression can vary but typically includes a combination of psychotherapy and medication. Cognitive-behavioral therapy and interpersonal therapy are common psychotherapeutic approaches. Antidepressant medications, such as selective serotonin reuptake inhibitors and serotonin-norepinephrine reuptake inhibitors (SNRIs), are often prescribed. In severe cases, treatments like electroconvulsive therapy or transcranial magnetic stimulations may be considered [17,18,19].

Although current methods of treatment are quite effective and widespread, all of them have downsides that should not be downplayed. The influence that REM sleep and depression have on each other is an avenue that many experts have explored in elaborating new and innovative diagnosis and treatment options. The present study was designed with the following main objective in mind: to assess sleep architecture—specifically REM sleep as a proportion of total sleep, a “REM sleep coefficient”—and correlate this coefficient with potential depressive symptoms in otherwise undiagnosed patients in order to better understand how alterations in sleep patterns, particularly in REM sleep, can serve as early indicators of depressive tendencies in individuals who have not been clinically diagnosed. This understanding could pave the way for early intervention strategies and improve mental health outcomes, employing new and advanced technologies that continuously emerge and get refined over time.

## 2. Materials and Methods

### 2.1. Participants

In total, 105 individuals participated, consisting of 44 men and 61 women. Each participant was over the age of 18, with the age distribution as follows: 27 were between 18 and 25 years, 36 were 26–30 years old, 30 were 31–40 years old, 7 were between 41 and 50 years, and 5 were between 51 and 60 years old. All participants had permanent residency in Romania at the time of completing the online survey. A significant majority of the respondents, 84 in total, resided in urban areas, while a minority of 21 lived in rural settings. The educational backgrounds of the participants showed diversity; 36 held a bachelor’s degree, 45 had completed postgraduate education, and 24 were high school graduates. In terms of marital status, a larger portion of the participants were unmarried (*n* = 63), followed by those in cohabitation (*n* = 22), and married individuals (*n* = 20). Professional status varied among the participants, with the majority being employed (*n* = 65), accompanied by freelancers (*n* = 15), entrepreneurs (*n* = 10), students (*n* = 12), and three unemployed individuals.

### 2.2. Procedure

A prospective study was conducted by researchers from the “Iuliu Hatieganu” University of Medicine and Pharmacy, Romania. Between October 2023 and March 2024, the participants’ sleep architecture and their self-perceived depressive symptoms were assessed by our team. For the participant enrollment, a one-time online survey based on Google Forms was shared through platforms of social media and via email chains. A copy of this survey and an Excel spreadsheet containing all demographic and sign-up information is available on request.

The anonymized online questionnaire had an approximate completion time of 5 min and contained 2 parts: a General Data Protection Regulation (GDPR) statement and a section dedicated to questions related to demographic data. The inclusion criteria were age, knowledge of the English language and the possession of an Apple Watch device and an iPhone smartphone (Apple Inc., Cupertino, CA, USA), both compatible to the proprietary mobile application used in this study (described more extensively in the following sections). Compatibility with the proprietary app was solely tied to the operating system version installed on the respective devices (iOS 17, watchOS 10), with no other restrictions. Therefore, a variety of iPhone models and Apple Watch models were included, with no perceived impact on the data collection itself. In line with the grant agreement through which this study was funded, several Apple Watch units were acquired, to be loaned to prospective participants with less financial capabilities, thus also broadening the inclusivity of our research. The exclusion criteria were as follows: the refusal or incapability of giving informed consent, comorbid sleep illnesses (such as narcolepsy or chronic insomnia), or any other relevant comorbid chronic illnesses (e.g., cardiovascular or pulmonary diseases).

Every participant was provided with a detailed written overview of the research and consented to participate after being fully informed. The secure and confidential handling of all data was ensured, in compliance with the General Data Protection Regulation (GDPR). Approval for this study was granted by the “Iuliu Hatieganu” University of Medicine and Pharmacy Cluj-Napoca, Romania’s Ethical Committee. The privacy of the respondents was and will be upheld at all stages.

For the purposes of this study, the research team made use of a proprietary algorithm and mobile application, based on the Apple Health application, in order to detect and quantify REM sleep stages. The algorithm, based on an AI (Artificial Intelligence) framework, inferences on real-time input data from a smartwatch and can be trained using multiple dataset sources. The architecture built around this concept facilitates the idea of scalability and further integration of this engine into more advanced future models that could potentially assist in a more sophisticated sleep analysis. The foundational element of the AI models is the comprehensive dataset collected in real sleep scenarios. The data, collated from both scientific repositories and our clinical study, are stored securely in AWS RDS (Amazon Relational Database Service).

Heart rate variability and tri-axial motion data are the main sensory inputs collected from smartwatches. Along with the aforementioned data, there are records of temporal information and sinusoidal patterns pertaining to circadian rhythms that form part of the additional features of the analysis. The data subsets are organized in real-time by user and date to create consistent sleeping sessions for future analysis. These sessions are further directly matched with the morning customary predictions provided by the smartwatch provider (Apple Inc., Cupertino, CA, USA). The deployed AI models receive 10 min segments of this physiological data. To refine the AI’s predictive capabilities, the data undergo a few stages of preprocessing. These objective metrics can be correlated with supplementary subjective inputs derived from user-completed questionnaires assessing depression and demographic variables.

Our team employed AWS SageMaker for the model training environment, allowing for a scalable and efficient computational workflow. The two-tiered classification system first determines the sleep state (asleep or awake) and subsequently identifies REM sleep phases. Models are trained using a blend of algorithmic approaches. Among the algorithms tested were logistic regression, random forest, k-nearest neighbors, ridge regression techniques, and neural networks. Hyperparameter-tuning proved that neural networks would emerge as best-suited for our classification tasks, following cross validation to avoid overfitting. The evaluations our models passed to achieve robust classification accuracy include standard statistical measures such as precision, recall, and F1 score, derived from a confusion matrix of the predicted sleep stages against the ground truth. The AI models’ performance is regularly evaluated against benchmark datasets and continuously refined. The AWS tools are used for the continuous monitoring of performance. Re-trainings also occur recurrently to leverage all the newly created data throughout a study.

Once included in this study, the participants spent 30 nights of sleep wearing the Apple Watch while the proprietary mobile application, developed for the purposes of this research, was running. Throughout this study, using the Apple Health app and the proprietary app, sleep data were collected by the research team, such as the timing of the first REM sleep phase in a sleep session, the total REM sleep phases, the total REM sleep duration, and the total sleep duration. The participants were administered the Beck Depression Inventory (BDI) through the same proprietary mobile phone app at four critical junctures: upon inclusion in this study (Day 0) and subsequently on Days 10, 20, and 30. The BDI is a widely used instrument for assessing the severity of depression in individuals. It consists of 21 questions, each designed to assess a specific symptom or attitude related to depression. The scoring system ranges from 0 to 63, with higher scores indicating more severe depressive symptoms. This self-report tool has been validated in numerous studies and is recognized for its reliability and validity in measuring the severity of depressive symptoms [20].

Upon the completion of data collection, all data were compiled in one table, containing a user ID (an approach compliant with GDPR, ensuring anonymization), the average BDI score across all 4 instances (“BDI” variable), and the average REM sleep duration percentage out of the total sleep duration (“REM” variable).

## 3. Results

### 3.1. Descriptive Statistics

This study encompassed a total of 105 participants, yielding data on both the Beck Depression Inventory (BDI) score averages and the average REM sleep duration percentages out of the total sleep duration (named from here on, “REM sleep coefficient”). The global average in the BDI scores across the participants was 23.71 (SD = 9.93, median = 24), with a range from 5 to 48. The participants exhibited an average REM sleep coefficient of 23.33 (SD = 6.56, median = 22.87), with values ranging from 10.1 to 40.26. A detailed table, containing (anonymized) data collected in our study, has been added to the Supplementary Materials.

### 3.2. Correlation Analysis

An outlier analysis was conducted using the Interquartile Range (IQR) method. No outliers were detected in either the BDI or REM variables. This absence of significant outliers simplified the analysis, allowing us to focus on the core distribution of the data without the need for adjustments or data transformations.

In assessing the distributional properties of the BDI and REM variables within our dataset, we employed both the Kolmogorov–Smirnov and Shapiro–Wilk tests to evaluate the assumption of normality. The Kolmogorov–Smirnov test yielded *p* values of 0.509 for BDI and 0.836 for REM, indicating a failure to reject the null hypothesis of normality for both variables. Similarly, the Shapiro–Wilk test, a more powerful test for normality, given the characteristics of our cohort, confirmed these findings (*p* = 0.085 for BDI and 0.147 for REM). These results suggest that both the BDI scores and the REM coefficients can be assumed to follow a normal distribution within the context of this study.

**Figure 1:**
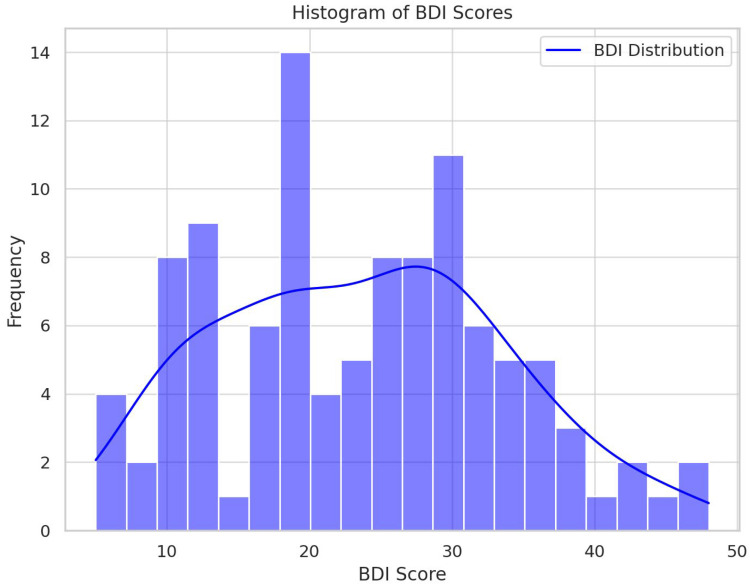
Histograms of BDI score averages.

**Figure 2:**
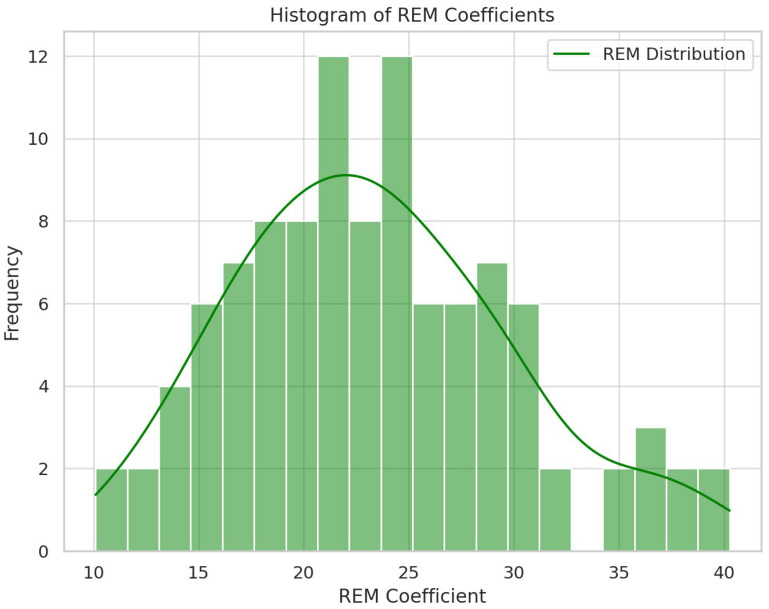
Histogram of REM sleep coefficients.

**Figure 3:**
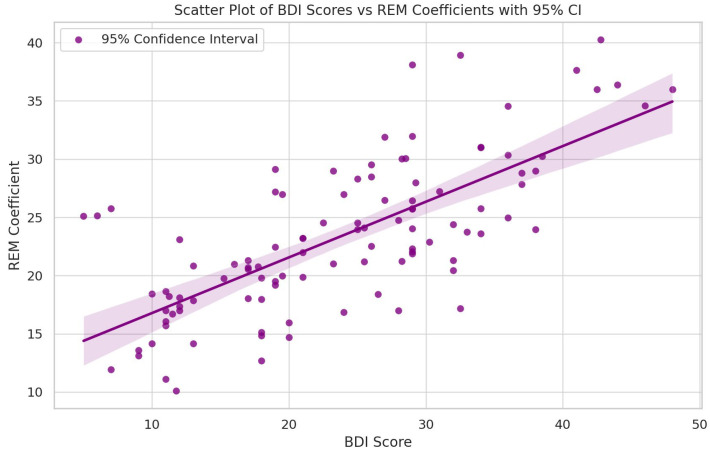
Scatter plot of BDI score averages vs. REM sleep coefficients.

Descriptive graphs—histograms and a scatter plot with a fit line with a 95% mean confidence interval—are shown below (Figure 1, Figure 2 and Figure 3).

**Figure 4:**
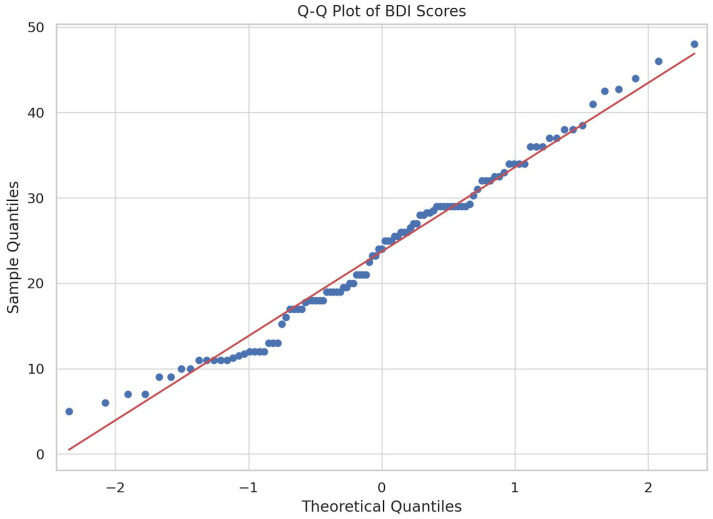
Q–Q plot of BDI score averages.

**Figure 5:**
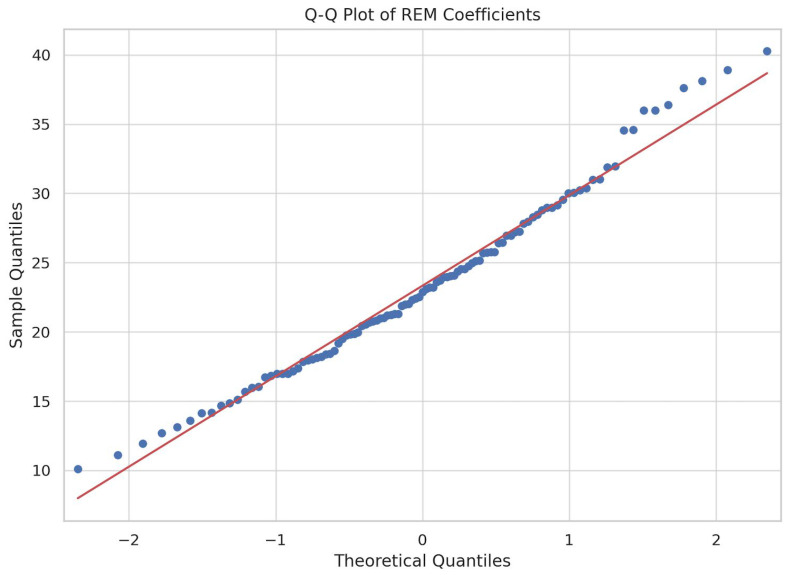
Q–Q plot of REM sleep coefficients.

To further assess the normality of our data, Quantile–Quantile (Q–Q) plots were constructed for both the BDI and REM variables (Figure 4 and Figure 5). In accordance to the structure of a Q–Q plot, each circle represents an observed value in our dataset, while the red lines serve as references that represent the theoretical perfect normal distributions.

Given the degree of normality and the characteristics of our cohort, the initial results suggest a cautious approach towards determining correlation, and as such, we employed the use of Spearman’s correlation coefficients to explore the relationship between the two variables. The Spearman’s correlation coefficient is particularly suited for our analysis, as it does not necessarily rely on the assumption of a higher degree of normality in the data and is less sensitive to outliers, making it a robust choice given the nature of our dataset.

Upon calculation, the Spearman’s correlation coefficient between the BDI and REM variables was found to be 0.702 (*p* value < 0.001). This value indicates a positive correlation, suggesting that as the BDI scores increase, there is a tendency for the REM sleep coefficients to increase as well.

### 3.3. Linear Regression

We conducted a linear regression analysis to investigate the relationship between BDI (independent variable) and REM (dependent variable). This analysis utilized an Ordinary Least Squares (OLSs) regression model. The regression model revealed that for every unit increase in the BDI score average, there is an expected increase of approximately 0.478 units in the REM sleep coefficient, suggesting a positive linear relationship between these two variables. The constant (intercept) of the model was found to be 12.002, indicating the expected value of the REM sleep coefficients when the BDI score is zero.

An essential aspect of the model’s interpretability is its R-squared value, which was calculated to be 0.522. This indicates that around 52.2% of the variability in the REM sleep coefficients can be explained by the model. The statistical significance of the model was confirmed with a Prob (F-statistic) < 0.001, suggesting that the model is a good fit for the data.

To ensure the validity of our regression model, we examined key assumptions such as the normality of residuals and homoscedasticity. The Q–Q plot of residuals (Figure S1) was analyzed to check for normality, and the scatter plot of residuals against predicted values (Figure S2) was examined to assess homoscedasticity. These diagnostic plots are crucial for verifying that the assumptions underlying linear regression are met, ensuring the accuracy and reliability of our findings. The two figures, Figures S1 and S2, are available in the Supplementary Materials.

## 4. Discussion

In the interpretation of our findings, we observed a significant positive correlation between the proportion of REM sleep and higher Beck Depression Inventory (BDI) scores. This aligns with existing research that has highlighted the strong relationship between sleep alterations, particularly in REM sleep, and depression. For instance, studies by Tsuno et al. [21] and Palagini et al. [22] consistently reported altered sleep architectures in depressed patients, including a shortened REM latency, an increased REM sleep duration, and an increased REM density. These alterations have been proposed as potential biomarkers for depression. Our study builds upon these findings by quantitatively analyzing the correlation between REM sleep disturbances and depressive symptoms. This not only reaffirms the established links in the literature but also provides a more detailed understanding of the extent to which REM sleep alterations could be indicative of depressive disorders. The consistency of our results with prior research, such as that of Tsuno et al. [21] and Palagini et al. [22], suggests that REM sleep disturbances are not merely symptoms of depression but could play a contributory role in its pathophysiology.

Building on the discussion of REM sleep as a biomarker for depression, recent research supports the potential of specific REM sleep parameters as indicators of depressive disorders. Studies have identified that REM latency (RL) and REM density (RD) can serve as electrophysiological markers of depression. This is particularly relevant in drug-free and comorbid-free patients with unipolar depression, as highlighted in a systematic review and meta-analysis by Arıkan et al. [23]. Their findings indicate a shortened RL and an increased RD in patients with unipolar depression compared to controls, aligning with the research mentioned in the previous paragraph. This supports the notion that alterations in REM sleep could be pivotal in the pathophysiology of depression. Furthermore, Wichniak et al. provided an extensive overview of sleep changes in depression, underscoring the sensitivity of sleep as a biomarker for brain functioning [24]. They emphasized the value of sleep biomarkers in relation to the risk, exacerbation, and treatment outcomes of depression. Among various sleep parameters, an increased REM density and a diminished delta sleep ratio were identified as areas of special interest. Their research also underscores the importance of sleep studies as a tool in antidepressant drug development. Lastly, our recently published narrative review highlights REM sleep deprivation as a valuable approach for the treatment of depression [25]. All these studies collectively reinforce the emerging perspective that REM sleep parameters can be critical biomarkers in understanding and managing depression. Finally, although our study primarily focused on REM sleep as a biomarker for depression, the potential of incorporating a broader range of biomarkers should be considered for a more comprehensive understanding of the disorder. Biomarkers like heart rate variability, activity levels, and even speech patterns, if captured and analyzed effectively, could offer additional insights. These metrics, particularly when combined with REM sleep data, can provide a more holistic view of an individual’s mental health state. Incorporating a multifaceted approach in future research could enhance the accuracy and efficacy of depression diagnosis and monitoring.

Given that our study focuses on undiagnosed patients exhibiting depressive symptoms, the clinical implications are particularly intriguing. The observed REM sleep disturbances in this group suggest that these sleep patterns may serve as early indicators of depressive tendencies, even before clinical diagnosis. This could revolutionize early screening and intervention strategies, allowing for preemptive treatment approaches in individuals showing signs of depressive symptoms. The alterations in REM sleep patterns observed in our study, such as a shortened REM latency and an increased REM density, align with the findings of Palagini et al. [22]. Furthermore, the study by Cartwright et al. explored the possibility of early REM sleep changes as a compensatory mechanism in depression [26]. Their research indicates that certain REM sleep characteristics might not only be symptomatic but could also play a functional role in the mood regulation of individuals experiencing stress, such as in the context of a divorce. This perspective opens up intriguing possibilities for understanding the role of REM sleep in the early stages of depressive symptom development. The potential of using REM sleep patterns in early detection underscores the need for greater awareness and assessment of sleep quality in routine health evaluations, especially for individuals at risk of developing depression. These insights emphasize the need for incorporating sleep assessments into routine health screenings, especially for individuals showing early signs of depressive symptoms. By monitoring and analyzing REM sleep patterns, healthcare providers could potentially identify and intervene in depressive disorders at a much earlier stage, improving patient outcomes.

In the current study, we meticulously analyzed the qualitative and quantitative relationship between REM sleep and depressive symptoms. An essential aspect of our analysis was addressing the ambiguity in the normality of data distribution, which informed our choice between Spearman’s and Pearson’s correlation coefficients. Firstly, we assessed the normality of the distribution of our variables. This assessment is critical, as Pearson’s correlation requires a normal distribution of data. However, given the characteristics of our data and the tendency for overestimation in the case of Pearson’s correlation, we also considered Spearman’s correlation, which does not necessarily require the same assumptions. Spearman’s correlation, being a non-parametric measure, is particularly robust. It is also less sensitive to outliers and is suitable for ordinal data or when the relationship between variables is not linear [27]. Consequently, we employed only Spearman’s correlation to ensure a more precise, more conservative analysis. The use of Spearman’s correlation allowed us to capture any monotonic relationships between variables, irrespective of their distribution. However, relying solely on Spearman’s correlation does bring certain limitations. This approach may not fully capture linear relationships as effectively as Pearson’s method. Additionally, the use of a non-parametric test generally provides a more conservative estimate of correlation strength. The potential of a higher correlation through Pearson’s rho coefficient is undeniable but not needed, given the preliminary nature of our results and the pilot nature of this study.

Moreover, our study’s methodological approach inherently had limitations. The sample size and demographic characteristics of the participants (the possession of an iPhone and an Apple Watch—non-essential, costly devices—as an inclusion criterion skews sample demographic characteristics toward wealthier and more educated individuals) may limit the generalizability of our findings. Recognizing this limitation, we implemented a strategy to enhance inclusivity by loaning Apple Watch units to participants who were less financially equipped to afford such technology, as settled upon in the grant agreement establishing the terms and conditions for our research. This inclusive initiative was crucial, especially considering the study’s duration of six months, during which the participants were required to engage in the study protocol for a total of 30 nights. The availability of loaned units significantly increased the participation of a more economically diverse cohort, accounting for a substantial portion of our study’s participants. By integrating individuals from varying economic backgrounds, we could capture a wider array of sleep patterns and health indicators, enhancing the depth and relevance of our analysis and mitigating the risk of selection bias—ultimately producing results that are more representative for a diverse society.

Additionally, the observational nature of this study precludes the establishment of causality. Future research could benefit from an even larger and more diverse sample size, as well as experimental designs to better understand the causal relationships between REM sleep disturbances and depressive symptoms. Conducting controlled experiments, where variables can be manipulated and the effects observed in real-time, would allow researchers to establish causal connections with greater certainty. Longitudinal studies could provide deeper insights into the temporal dynamics between REM sleep disturbances and depressive symptoms. By tracking participants over extended periods, researchers can observe the progression of symptoms and identify potential critical windows for intervention. This longitudinal approach, combined with experimental methodologies, could uncover the mechanisms underlying the relationship between sleep and mental health, paving the way for targeted treatments and preventative strategies.

In considering the demographics of our study participants, it is essential to recognize that the sample largely comprised urban residents with access to an Apple Watch, indicating a certain level of economic affluence. Additionally, the majority of the participants possessed higher education degrees. These factors—urban residency, economic status, and educational background—may have implications for our findings. The urban environment can uniquely impact health behaviors and stress levels, potentially influencing both REM sleep and BDI scores. Similarly, higher education levels might correlate with better health literacy, affecting disease perception and management. Acknowledging these demographic biases is crucial, as they might limit the generalizability of our results. We emphasize the preliminary nature of our pilot study and the necessity for subsequent research with larger, more diverse participant groups, spanning various socio-economic backgrounds, educational levels, and living environments, to provide a more comprehensive understanding of the studied disorder in different societal strata.

A pivotal aspect of our methodology and potentially important concern would be the validity and reliability of our methods, regarding data collection and sleep stage prediction, as well as the hardware and platform choice. The model was trained on extensive data. The Apple Watch was used to collect raw biodata, including acceleration and heart rate measurements. Our custom-developed mobile application facilitated real-time data capture and transmission for analysis. The real-time sleep stage prediction is reliant upon the methodology presented in a previously published article [28]. The focus of our research is based on worldwide consumer-accessible hardware that has gone through medical device registration processes, such as the Apple Watch, which has already received FDA approval as a Class II medical device. This proves that the sensors and overall hardware in the Apple Watch can be trusted as a source of data, is replicable across populations, and is reliable for a sleep study. From a validity and reliability standpoint, one must be aware of the fact that sleep stage prediction based on wearable devices is limited to certain input data points and currently, as of February 2024, Apple’s own sleep stage analysis system is considered state of the art, at 76.6% accuracy when compared to an EEG device. For more information, a detailed table representing a comparison to an EEG headband, on which a paper was previously published [29], can be found among the Supplementary Materials (Table S1). Therefore, from this point of view, data collected by an Apple Watch is considered to be consistent and relevant across populations.

To the best of our knowledge, this is the first study that demonstrates the ability of a widely used consumer wearable device to estimate sleep stages using investigator-, as opposed to manufacturer-, developed algorithms and the generalizability of these algorithms to data collected by traditional methods. Compared to polysomnography, our neural network model, applied to Apple Watch-derived heart rates, motions, and a computed circadian estimate, demonstrated sleep/wake differentiations with 93% of sleep epochs scored correctly, 60% of true wake epochs scored correctly, and a REM–nonREM sleep stage differentiation accuracy of 72% [28]. For even further validation, the model was and still is continuously tested against external data from the MESA dataset [30,31]. For the purposes of this study, a subset of the data (188 subjects, chosen for computational feasibility), with co-recorded actigraphy and polysomnography data, was extracted and processed for use as an independent testing dataset. Testing against an external dataset confirmed the model’s effectiveness and applicability to broader populations, reinforcing its robustness and potential for widespread use in sleep analytics. All predictions are also validated through Apple’s state-of-the-art post-processing system, considering the previously mentioned accuracy of 76.6% when compared to an EEG device. As mentioned in the Methods section, the results were consistently replicated over multiple runs. The model was validated against polysomnography data using leave-one-out and Monte Carlo cross-validation methods. This rigorous validation process ensures our model’s reliability and generalizability across different datasets. Our machine learning models, including neural networks, logistic regression, k-nearest neighbors, and random forest classifiers, were compared for optimal performance. The neural network model was identified as the most accurate, particularly for sleep–wake classifications. The neural network model achieved an internal classification accuracy of approximately 90%, with a detailed breakdown of specificity and sensitivity for identifying wake and sleep epochs. This high level of accuracy demonstrates the model’s capability in accurately distinguishing between different sleep stages. This model is used as a first step, prior to triggering the NREM/REM classification, which has an accuracy of 72% (close to 76.6% of Apple’s state-of-the-art sleep stage prediction system, on an FDA-approved medical device).

As more and more people suffer from depression in an increasingly stressful and intense environment [32,33], there is an urgent need for research focus in the field. We propose several key areas for further exploration. Firstly, longitudinal studies are essential to understand the progression and temporal dynamics of how changes in REM sleep may lead to the development of depressive symptoms. This approach will enable a deeper understanding of the potential predictive nature of REM sleep alterations for depression. Additionally, it is imperative to establish causality through experimental designs. Studies could involve the intentional modulation of sleep patterns to observe the resultant effects on mood and mental health, providing direct insights into the causal relationship between REM sleep and depressive symptoms. Expanding research to include diverse populations is also crucial. Studies involving varied demographic groups, encompassing different ages, genders, and cultural backgrounds, will enhance the generalizability and applicability of our findings. Such diversity in research participants will allow for a more comprehensive understanding of the REM sleep–depression relationship across different segments of the population. Lastly, the exploration of intervention strategies that specifically target REM sleep could be revolutionary in the treatment of depression. Research in this area might include the development and testing of pharmacological and non-pharmacological interventions aimed at normalizing REM sleep patterns, thereby potentially mitigating depressive symptoms.

On a more general note, through recognizing the potential of wearable technology like the Apple Watch in mental health, we must consider its role in empowering individuals to monitor their own health. The Apple Watch enables users to track sleep patterns and other health metrics, acting as a tool for proactive health management. For instance, users can monitor changes in their sleep architecture, including REM sleep, and relate these changes to their mental well-being. Such personal health data can then be shared with healthcare providers, facilitating early diagnosis and personalized treatment strategies. This integration of technology into everyday health monitoring represents a significant shift towards patient-centered care, where individuals are actively involved in their health management and have the potential to collaborate more closely with their healthcare providers.

Finally, as discussed extensively, our current findings point to REM sleep having a potential role in the pathophysiology of depression, without definitively establishing a causal link between sleep architecture alterations and depression. This current study is merely an exploratory one, aiming to enrich the understanding of the involvement of REM sleep changes in affective disorders. Albeit promising, the results should always be interpreted in the greater context of each individual patient. The potential link that has been found through a positive correlation coefficient is only one of the very numerous factors to be taken into consideration in the complex network of managing depression. Further experimental studies in this pioneering field should be conducted, exploring the avenues of depression pathophysiology and any potential causal role that REM sleep disruptions play during the stages of the illness.

To the best of our knowledge, this is the first study to utilize the Apple Watch as a tool for measuring REM sleep patterns and correlating them with depressive symptoms. This innovative approach leverages the advanced technology of wearable devices to gain insights into the intricate relationship between sleep and mental health. Our pilot study represents an exploratory foray into the potential of using a commercial device like the Apple Watch to detect REM sleep and its correlation with depressive symptoms. We recognize that recommending such devices for the clinical assessment of depression requires caution. This study is intended as a preliminary investigation into the feasibility and potential of wearable technology in this field, not as a definitive endorsement of its clinical utility. The findings should be viewed as an initial step in a broader research agenda aimed at validating and refining digital health tools for mental health assessment.

## 5. Conclusions

In conclusion, our findings suggest a positive correlation between REM sleep disturbances and the severity of depressive symptoms, indicating the potential for REM sleep parameters to serve as biomarkers in depression diagnosis, and highlight the potential of consumer wearable devices, such as the Apple Watch, in the diagnosis and management of affective disorders. This research not only contributes to our understanding of the neurobiological underpinnings of depression but also opens avenues for novel therapeutic approaches targeting sleep architecture. Future studies should expand on these findings, exploring the causative links and the efficacy of sleep-focused treatments in depression management.

## Data Availability

A dataset containing relevant sleep data and BDI scores is present in the Supplementary Materials.

## Supplementary Materials

The following supporting information can be downloaded at: https://www.mdpi.com/article/10.3390/jpm14050519/s1, Dataset, Figure S1: Q–Q plot of residuals; Figure S2: Residuals vs. predicted values; Table S1: Apple Watch vs. EEG headband.
