# Supplementary figures and images for: Deciphering the Link: Correlating REM Sleep Patterns with Depressive Symptoms via Consumer Wearable Technology
Source: J Pers Med. 2024 May 14;14(5):519. doi: 10.3390/jpm14050519 (PMC11121981; doi:10.3390/jpm14050519)

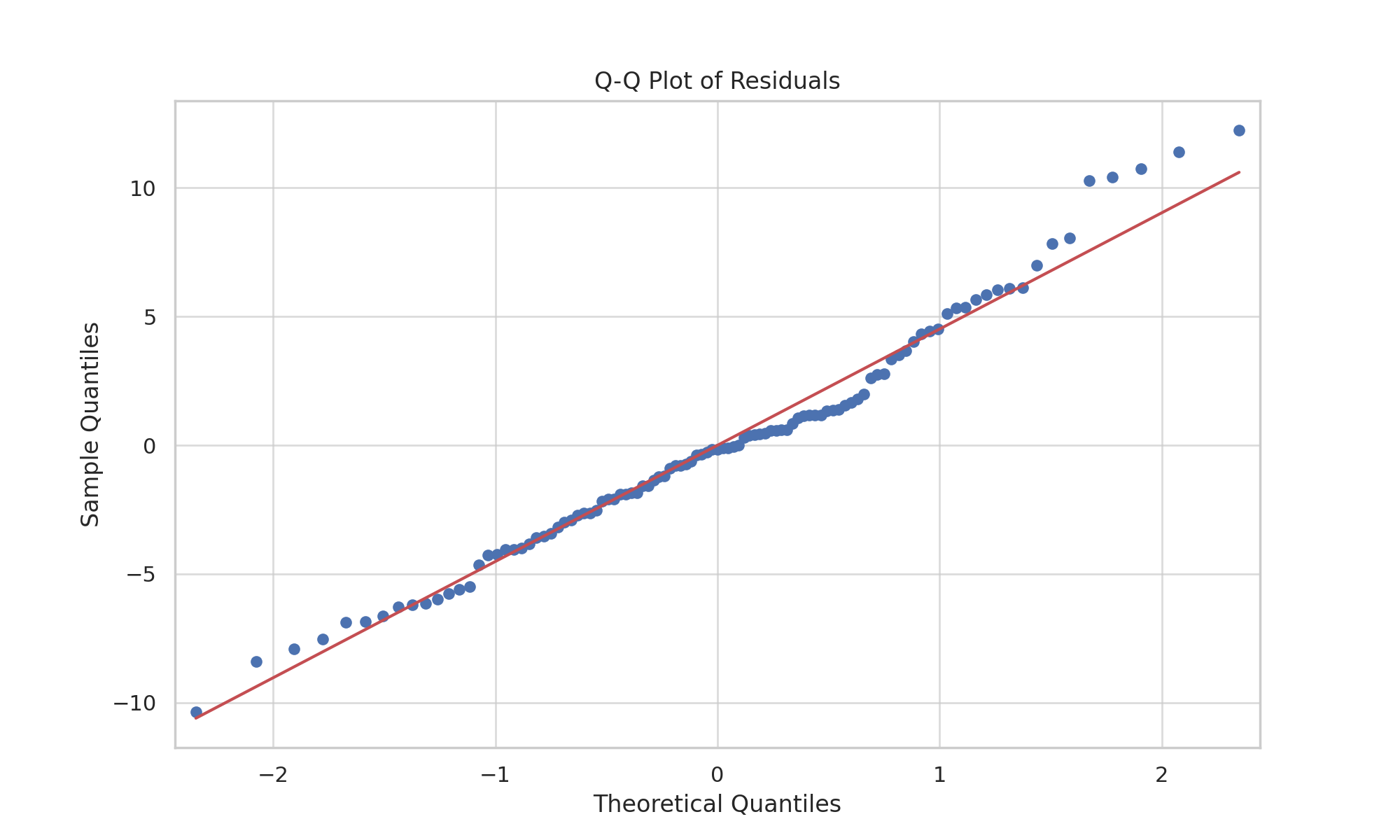

Supplement: Supplementary file 1 [file jpm-14-00519-s001.zip › Figure S1.png]

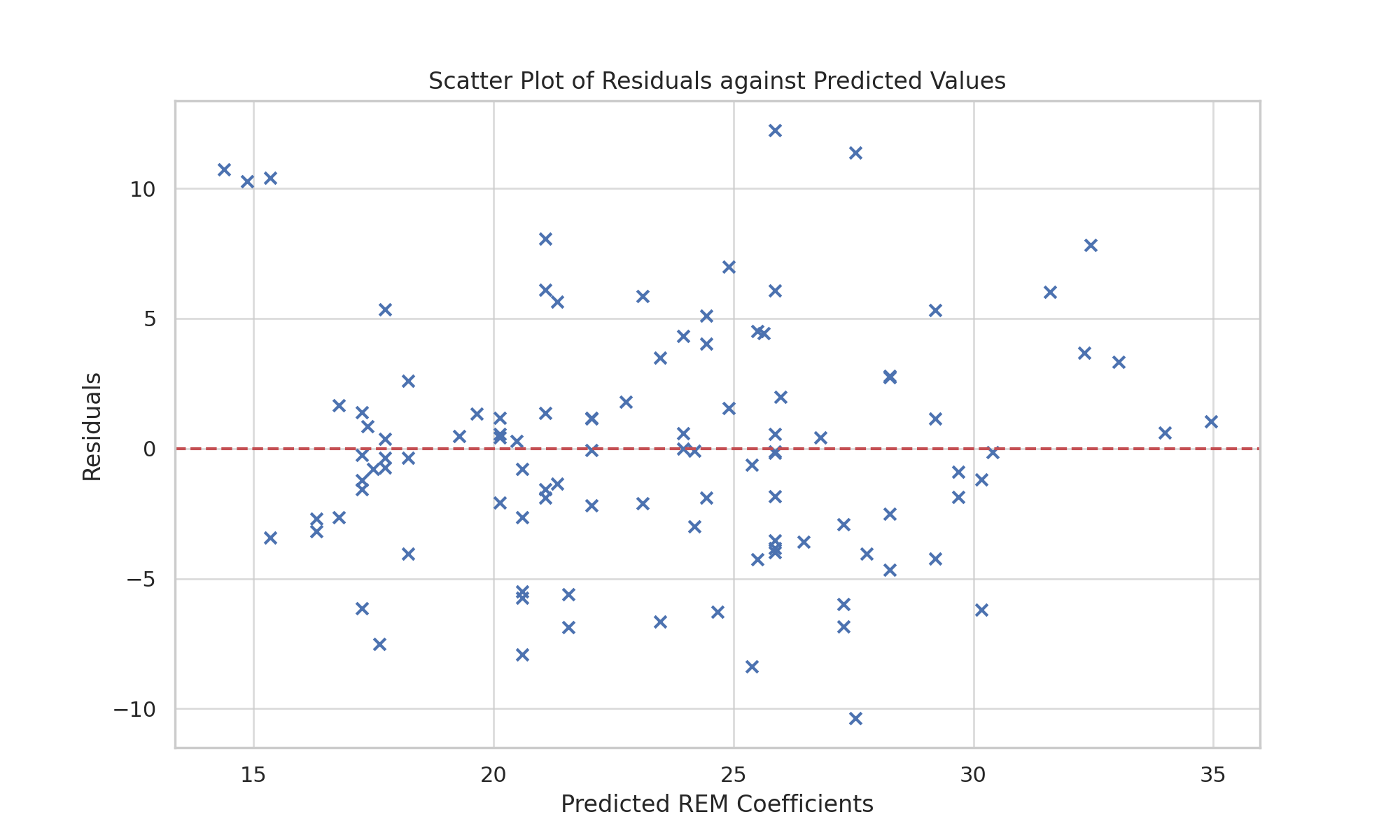

Supplement: Supplementary file 1 [file jpm-14-00519-s001.zip › Figure S2.png]

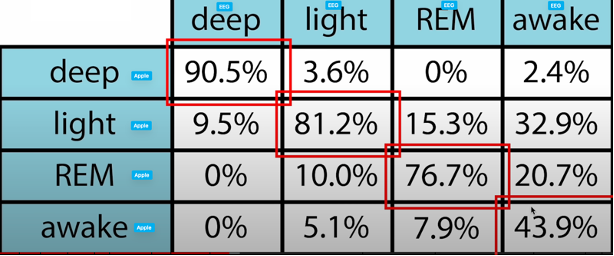

Supplement: Supplementary file 1 [file jpm-14-00519-s001.zip › Table S1.png]
